# Supplementary material for: Nanogap‐Engineerable Electromechanical System for Ultralow Power Memory
Source: Adv Sci (Weinh). 2017 Dec 3;5(2):1700588. doi: 10.1002/advs.201700588 (PMC5827012; doi:10.1002/advs.201700588)
Supplement: Supplementary file 1 — Supplementary [file ADVS-5-1700588-s001.pdf]

Copyright WILEY-VCH Verlag GmbH & Co. KGaA, 69469 Weinheim, Germany, 2017.

ADVANCED SCIENCE
Open Access

Supporting Information

for *Adv. Sci.,* DOI: 10.1002/advs.201700588

Nanogap-Engineerable Electromechanical System for Ultralow Power Memory

Jian Zhang, Ya Deng, Xiao Hu, Jean Pierre Nshimiyimana, Siyu Liu, Xiannian Chi, Pei Wu, Fengliang Dong, Peipei Chen, Weiguo Chu, Haiqing Zhou,* and Lianfeng Sun**

Copyright WILEY-VCH Verlag GmbH & Co. KGaA, 69469 Weinheim, Germany, 2016.

Supporting Information

Nanogap-engineerable electromechanical system for ultralow power memory

Jian Zhang, Ya Deng, Xiao Hu, Jean Pierre Nshimiyimana, Siyu Liu, Xiannian Chi, Pei Wu, Fengliang Dong, Peipei Chen, Weiguo Chu, Haiqing Zhou*, and Lianfeng Sun**

S1. The fabrication details of the SWNT devices

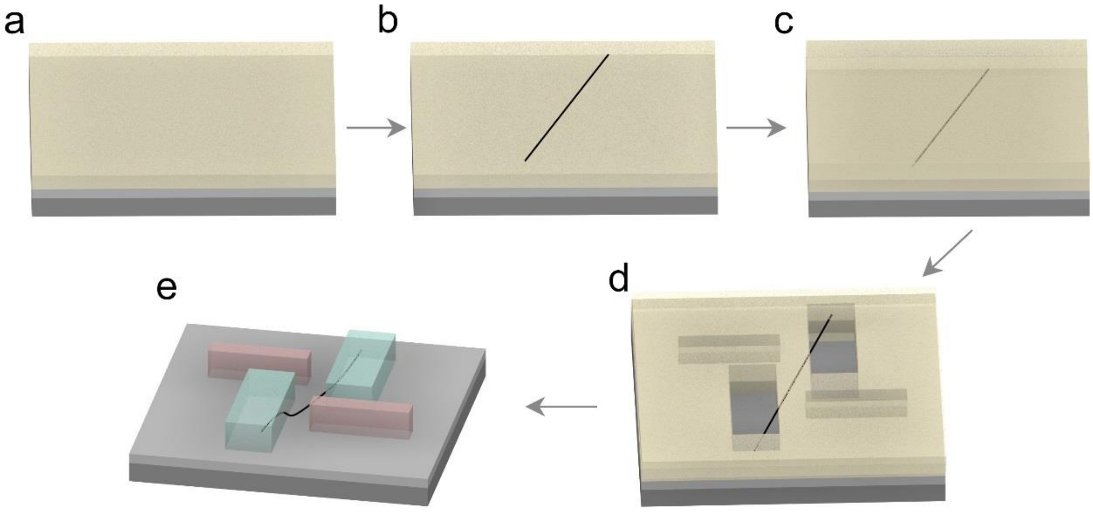

Figure S1. The fabrication details of the SWNT devices. a) Spinning a layer of polymethylmethacrylate (PMMA, thickness of 100 nm) on a doped silicon with 300 nm SiO_2. (b) Deposition of individual SWNT on the PMMA. c) Spinning another layer of PMMA (200 nm) on the surface of the samples. d) Four electrodes (S, D, G1 and G2) are patterned according to the position and orientation of the SWNT with EBL. e) Evaporation of nickel on the device, followed by lift-off process.

S2. The characterization of the suspended section of SWNT

To check the suspended length of the SWNT with SEM, seven SWNT devices with larger deflection angle (> 60°) were fabricated. The suspended section of SWNT can be distinguished by SEM when the electron beam was focused on the surface of the electrode as shown in Figure S2a. The projected length (*l*) of these seven devices is measured and the average value is about 226 nm (Table S1). Because all the fabrication parameters are the same with that of the SWNT devices with 10°deflection angle, therefore this average value of the projected length (226 nm) is also applicable for the latter one. When there is an angle of 10° between the SWNTs and the electrodes, the maximum distance (*d*) between the suspended SWNTs and the electrodes is below 40 nm ($d = l \sin \alpha$), as shown in Figure S2b. A sub-40 nm-wide region is created between the suspended section of SWNT and the electrode, which provides a reliable confinement to create nanogap by electroburning method.

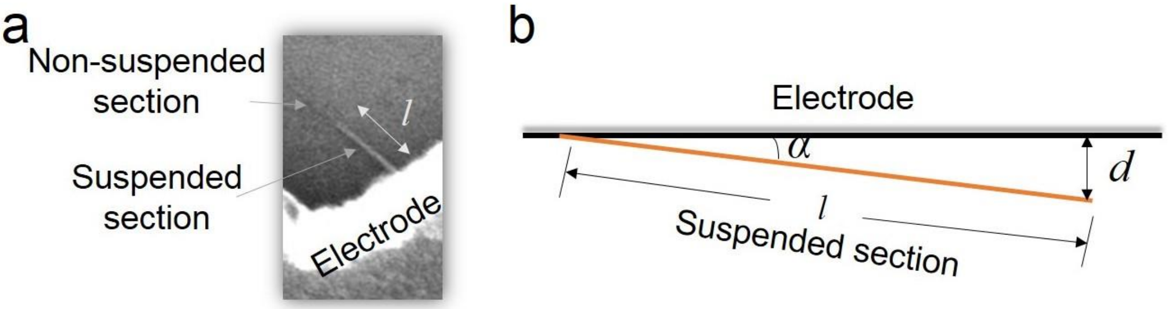

Figure S2. a) SEM image of the SWNT device with larger angle deflection. b) Geometric model of the junction between SWNT and electrode.

Table S1. The values of *l* for seven devices

**devices**	1	2	3	4	5	6	7	**average**
***l* (nm)**	188	201	169	320	205	236	264	**226**

S3. The fabrication and operation of a semiconducting SWNT switching memory in ambient conditions

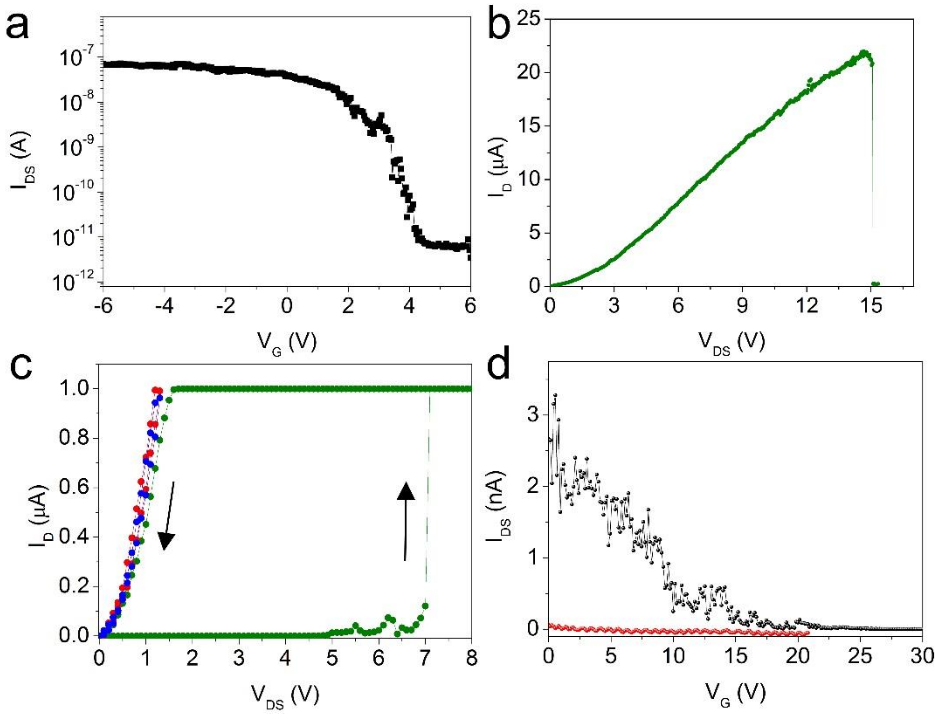

Figure S3. The fabrication and operation of a semiconducting SWNT memory in ambient conditions. a) Drain current versus gate voltage V_G (at V_{DS}=100 mV) of the semiconducting SWNT device at room temperature. The V_G was applied at the backgate of doped silicon, which the thickness of the SiO_2 is 300 nm. b) Typical *I*-*V* curve during electroburning of SWNT device in ambient conditions. A sharp decrease of current is observed at 15.1 V, indicating electrical breakdown of SWNT (resistance: ~GΩ). The drain voltage was interrupted immediately when the electrical breakdown happened. c) *I*-*V* characteristics of the semiconducting SWNT memory device switching from OFF to ON state. After electrical breakdown, the resistance of the device is high (~GΩ), indicating the state of OFF. When the voltage is swept up and reaches a critical value (switching ON voltage at 7 V), the resistance of the device becomes low (<1 MΩ), suggesting the state of ON. Then the ON state can be kept as indicated by the successive sweeping of voltage (red and blue curves), indicating the nonvolatility of the memory device. d) Switching of ON to OFF of the semiconducting SWNT memory device by applying a voltage to the gate electrode (V_G). Here a small bias (10

mV) is applied between the drain and the source electrodes and the corresponding current I_D is monitored as the voltage on the gate electrode is swept up. Unlike the switching curve of metallic SWNT device, the resistance was decreased to a high value gradually and we didn't observe a critical switching OFF voltage clearly, mainly because the electrical property of the semiconducting SWNT. However, after the first sweeping into 30V, the gate voltage was applied again. This time the resistance was very high (~ GΩ), indicating that the SWNT memory device has been switched into OFF state at the first sweeping.

S4. Details of the Simmons fitting procedure

The Simmons model describes tunneling through a potential barrier of arbitrary shape by the following equation:[36]

$$J = J_0\left[\frac{\bar{\emptyset}\exp(\frac{-2d\beta}{h}\sqrt{2m}\sqrt{\bar{\emptyset}})}{J_L/J_0} - \frac{(\bar{\emptyset}+eV)\exp(\frac{-2d\beta}{h}\sqrt{2m}\sqrt{\bar{\emptyset}+eV})}{J_R/J_0}\right] \qquad (1)$$

with $J_0 = \frac{e}{4\pi^2 h d^2 \beta^2}$, where J is the current density, e is the elementary charge, h is the reduced Planck's constant, $\bar{\emptyset}$ is the mean barrier height, m is the electron mass, V is the potential applied and β is a correction factor which is 1 to a good approximation. We simplify the Simmons equation with the assumption that carbon nanotube and nickel have same work functions (~4.6 eV).

We assume a circular barrier with $V < \emptyset_b/e$ (Figure S4), where $\emptyset_b$ is the height of the rectangular barrier. In this case $\bar{\emptyset} = \emptyset_b - eV/2$, using equation (1) we can calculate the current I as follows:

$$I = \frac{Ae}{4\pi^2 h d^2}\left[\left(\emptyset_b - \frac{eV}{2}\right)\exp\left(\frac{-2d}{h}\sqrt{2m}\sqrt{\emptyset_b - \frac{eV}{2}}\right) - \left(\emptyset_b + \frac{eV}{2}\right)\exp\left(\frac{-2d}{h}\sqrt{2m}\sqrt{\emptyset_b + \frac{eV}{2}}\right)\right] \qquad (2)$$

where A is the area of the junction, approximated to the sectional area of SWNT (diameter ~1.6 nm). Thus the *I*-*V* curves of the nanogap were fit to equation (2) appropriately. As shown in Figure 2e in the main text, the values deduced from this fit are as follow: gap size d=1.94 nm and gap barrier Φ_b=0.5 eV. In our devices, the gap size ranged between 1 to 5 nm.

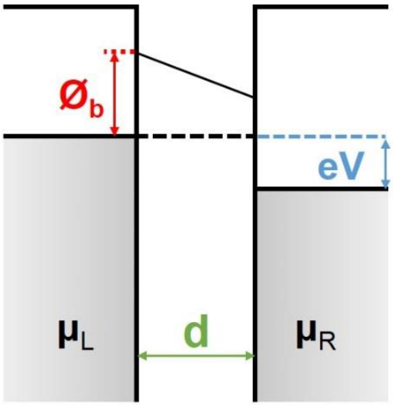

Figure S4. Potential barrier with thickness d between SWNT and nickel electrode, μ_L and μ_R are the chemical potentials. $V < Ø_b/e$

S5. Stable SET operations of SWNT memory devices

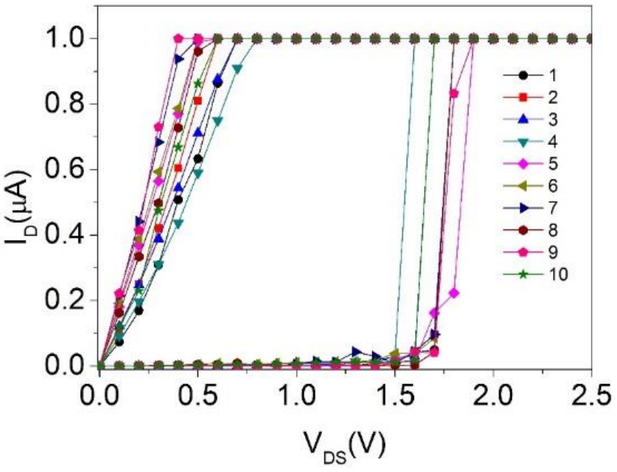

Figure S5. *I*-*V* characteristics of memory device S1 switching from OFF to ON state for 10 times. The switching ON voltages are ranging from 1.5 V ~ 1.8V, showing stable SET operations of the memory device.

S6. Calculation of the capacitor made of the SWNT and electrodes

Using the following model and equation of the capacitance of an unparallel-plate capacitor, the capacitor made of the SWNT and the drain electrode can be obtained:

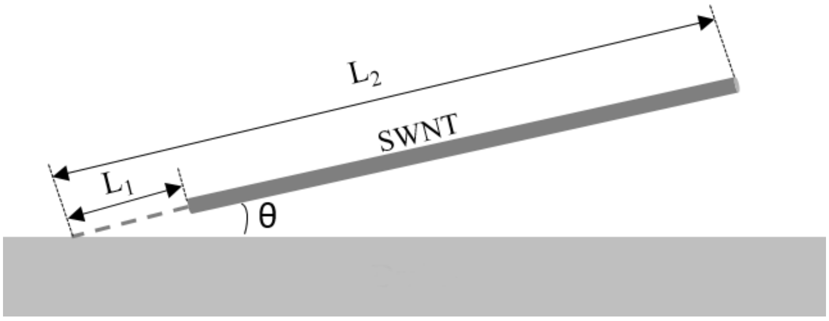

Figure S6. Geometric model of unparallel-plate capacitor made by SWNT and drain electrodes.

Based on the model of unparallel-plate capacitor, the capacitance can be calculated by the equation:[39]

$$C = \frac{\varepsilon_0 R}{\theta} \ln \frac{L_2}{L_1}, \qquad (1)$$

where ε_0 is the vacuum permittivity (8.85×10^{-12} F/m), R is the width of the plate (diameter of the SWNT, 1.6 nm)[27], θ is the angle between SWNT and the drain electrode (10 °), L_1 and L_2 are the lengths shown above (for the device in the main text, the values are 30 nm and 2600 nm, respectively). The capacitor C is estimated to be 3.6×10^{-19} F.

REFERENCES

[39] Y. Xiang, *Commun. Nonlinear Sci.* **2007**, *12*, 652.
